# The Benefits of Flavonoids in Diabetic Retinopathy

**DOI:** 10.3390/nu12103169

**Published:** 2020-10-16

**Authors:** Ana L. Matos, Diogo F. Bruno, António F. Ambrósio, Paulo F. Santos

**Affiliations:** 1 Department of Life Sciences, University of Coimbra, Calçada Martim de Freitas, 3000-456 Coimbra, Portugal; anna.matos1999@gmail.com (A.L.M.); diogobrunoa13@gmail.com (D.F.B.); 2 Coimbra Institute for Clinical and Biomedical Research (iCBR), Faculty of Medicine, University of Coimbra, 3000-548 Coimbra, Portugal; afambrosio@fmed.uc.pt; 3 Center for Innovative Biomedicine and Biotechnology (CIBB), University of Coimbra, 3000-548 Coimbra, Portugal; 4 Clinical Academic Center of Coimbra (CACC), 3004-561 Coimbra, Portugal; 5 Association for Innovation and Biomedical Research on Light and Image (AIBILI), 3000-548 Coimbra, Portugal

**Keywords:** diabetic retinopathy, flavonoids, neuroprotection, oxidative stress, inflammation, retina

## Abstract

Diabetic retinopathy (DR), one of the most common complications of diabetes, is the leading cause of legal blindness among adults of working age in developed countries. After 20 years of diabetes, almost all patients suffering from type I diabetes mellitus and about 60% of type II diabetics have DR. Several studies have tried to identify drugs and therapies to treat DR though little attention has been given to flavonoids, one type of polyphenols, which can be found in high levels mainly in fruits and vegetables, but also in other foods such as grains, cocoa, green tea or even in red wine. Flavonoids have anti-inflammatory, antioxidant and antiviral effects. Since it is known that diabetes induces oxidative stress and inflammation in the retina leading to neuronal death in the early stages of the disease, the use of these compounds can prove to be beneficial in the prevention or treatment of DR. In this review, we summarize the molecular and cellular effects of flavonoids in the diabetic retina.

## 1. Diabetes and Diabetic Retinopathy

Diabetes mellitus is a group of metabolic diseases characterized by chronic hyperglycemia resulting from defects in insulin secretion, insulin action or both, leading to various serious damaging consequences such as heart attacks, blindness, kidney failure, leg amputation and stroke.

Diabetes contributes to several visual impairments such as cataracts, glaucoma and, most importantly, diabetic retinopathy, one of the most common complications of diabetes [1]. Although there are significant differences between various regions of the globe, a recent study calculates the global prevalence of DR at 27% [2]. This means that of the 463 million diabetic adults as of 2019, 125 million have DR. Furthermore, the already disturbing number of diabetic adults is estimated to increase even further, reaching 700 million in the year 2045 [3] in which, assuming the prevalence of DR among diabetic adults is maintained, will originate approximately 189 million cases of DR worldwide. After 20 years of diabetes, nearly all patients with type 1 diabetes (T1D) and 60% of patients with type 2 diabetes (T2D) have diabetic retinopathy [4]. A recent case–control study showcased how DR influences people’s quality of life and life satisfaction, using two groups of T2DM patients, one with 70 patients with DR and another group with 70 patients without DR. Patients with DR had significantly worse scores in all scales related to quality of life and life satisfaction compared to patients without DR [5].

Risk factors for DR can be classified in two main categories: non-modifiable and modifiable risk factors. Non-modifiable risk factors associated with DR include duration of diabetes, renal disease [6], puberty and pregnancy [7] as it is estimated that diabetes affects 17% of pregnancies worldwide [8]. Modifiable risk factors associated with DR include obesity, smoking, hyperglycemia, hypertension and dyslipidemia [7,9]. All these factors contribute to the development of diabetes and ultimately increase the risk of DR. Other eye complications of diabetes, such as cataracts, also might contribute to increase the risk of developing DR. Cataract surgery in diabetic patients who do not have DR increases the risk of developing non-proliferative DR (NPDR) when receiving the surgery [10].

## 2. Pathogenesis of Diabetic Retinopathy

Diabetic retinopathy is characterized by an inflammatory process that involves anatomic and functional changes in the retina [11,12]. Structural and physiological features make the retina a tissue of high metabolic demands [13]. Accordingly, to attain adequate blood supply, the primate retina is nourished by two discrete circulatory systems: the choroidal and the retinal vasculature. Within the choroid, a dense network of wide-lumen capillaries called the choriocapillaris provides oxygen and nourishment to the highly metabolic photoreceptors, the pigment epithelium and also the outer plexiform layer [14]. The retinal vasculature, visible by fundus examination, provides the remaining blood flow to the inner retina. The retinal vessels are not fenestrated, unlike the choriocapillaris, and the presence of tight junctions between endothelial cells form a barrier restricting the movement of ions and large molecules into and out of the retinal parenchyma. These specialized bonds are the foundation of the blood–retinal barrier (BRB), which is essential for retinal homeostasis [15]. Similar to the blood–brain barrier, the BRB not only controls the exchange of materials between the blood and the retina, but also ensures the immunologically privileged environment characteristic of the CNS [16].

DR has classically been considered as a disease of the retinal microvasculature, starting with a progressive alteration of the BRB permeability, vascular occlusion, formation of macular edema and tissue ischemia. DR may then progress to a more damaging phase with proliferation of new blood vessels, increased ischemia and detachment of the retina. The first stage of DR, NPDR, is diagnosed when microvascular lesions are detected in fundus examination, usually microaneurysms resulting from dilation of capillary walls [17]. As the damage progresses, other signs become apparent during eye examination, such as intraretinal hemorrhages, hard exudates and lipid deposits formed upon BRB impairment. These signs indicate increased levels of vascular permeability which, as the disease progresses, causes macular edema (DME). This accumulation of subretinal and intraretinal fluids alters the structure and function of the macula and can lead to severe visual loss if untreated. In the most advanced non-proliferative stage, capillary occlusions and fine irregular vessels are present in examinations, as well as the dilation of major retinal vessels in association with ischemic regions. The evidence of neovascularization marks the final stage of DR, known as proliferative DR (PDR), where the hypoperfusion caused by capillary occlusion induces the formation of new blood vessels, mainly through the release of vascular endothelial growth factor (VEGF) by cells under hypoxic conditions [18,19], most likely in an attempt to revascularize the ischemic tissue. While loss of visual acuity in DR results mainly from macular edema [20], the resulting ischemia and the intraretinal and vitreous hemorrhages can also impair central vision, while leaving peripheral vision unaffected. In the more advanced cases of DR, the retinal detachment caused by extensive neovascularization can cause severe, irreversible vision loss.

While it is unequivocal that diabetes-induced microvascular alterations lead to pathologies of grave threat to vision, the conventional view of DR as mainly a microangiopathy is being currently challenged. It is now clear that retinal neurodegeneration and inflammation occurs very early in the course of diabetes, even in the absence of clinically visible microvascular abnormalities [12,21,22]. Structural neurodegenerative changes such as neural apoptosis, loss of ganglion cell bodies, glial reactivity and reduction in thickness of the inner retinal layers have been described [23,24,25]. This loss of neural tissue is in line with previous functional studies showing neuroretinal deficits in patients with diabetes including electroretinogram abnormalities, loss of dark adaptation and contrast sensitivity and color vision disturbances [24,25].

Many of these neurodegenerative changes are promoted by the chronic inflammatory environment observed in diabetic retinas. Increased levels of proinflammatory cytokines, such as tumor necrosis factor α (TNF-α) and interleukin-1β (IL-1β), were found in the retina and in the vitreous of patients with DR [26,27,28]. Interleukin converting enzyme/caspase 1, the enzyme responsible for the production of biological active IL-1β [29], is activated in diabetic retinas [30,31] and when IL-1β is administered, vascular permeability increases, which appears to be mediated by leukocyte adhesion, nuclear factor kappa-B (NF-κB) activation and retinal capillary cell death [32]. TNF-α is the prototypical member of a family of cytokines that induces apoptosis, differentiation, cell activation and inflammation. TNF-α is found in the extracellular matrix, endothelium and vessel walls of fibrovascular tissue in PDR. Blockade of the TNF-α pathway with antibodies against TNF-α receptor 1 (TNFR1), a TNF-α receptor associated with cell death, prevented not only the retinal vascular alterations of diabetes [33], but also the death of retinal neurons induced by elevated glucose [34]. Although in the retina these proinflammatory cytokines can be released by various cell types, such as endothelial cells, macroglial cells or even neurons [35,36,37], microglia and infiltrant monocytes are their principal producers in diabetic retinas [27]. The increase in the number of these cells in the subretinal space of diabetic retinas [38] is facilitated by the pores formed in retinal pigment epithelium (RPE).

Reactive oxygen species (ROS, e.g., hydroxyl radical (OH-)) and reactive nitrogen species (RNS, e.g., nitric oxide, NO) are well recognized for having a dual role as both beneficial and harmful species. Low to moderate levels of ROS and RNS are needed for physiological activities as they act as messengers in redox signaling to promote cell differentiation, proliferation, metabolism, immune system control and regulation and vascular restoration. To manage ROS and RNS levels, cells use enzymatic and nonenzymatic antioxidant defense systems. Oxidative stress, arising as a result of an imbalance between ROS and RNS production and antioxidant defenses, is associated with damage to a wide range of biological macromolecules such as lipids, carbohydrates, proteins and nucleic acids [39] and is involved in the induction of several pathologies as diabetes mellitus, neurodegenerative diseases and several eye diseases including age-related macular degeneration, glaucoma and DR [40]. ROS promote nuclear factor-κB (NF-κB) production which, in turn, mediates VEGF expression, and at the same time, it is activated by VEGF and translocated to the nucleus to promote the expression of proinflammatory mediators such as Intercellular Adhesion Molecule 1 (ICAM-1), vascular cell adhesion molecule-1 (VCAM-1), cyclooxygenase-2 (COX-2) and monocyte chemotactic protein 1 (MCP-1) [41]. Diabetes and ROS induce local hypoxia with upregulated mRNA expression levels of proapoptotic proteins, hypoxia-inducible factor 1α (HIF-1α) and 5′-adenosine monophosphate-activated protein kinase (AMPK) phosphorylation, which mediate cell death. Protein kinase C (PKC) is a family of cAMP-dependent protein kinases with multiple isoforms. PKC regulation by a rise in oxidative and nitrosative stress can contribute to the redox mechanism-mediated signaling events of the DR pathogenesis in many cellular functions, such as cell survival, migration, growth, proliferation and apoptosis [42]. PKC-δ is implicated in the apoptosis of capillary cells by increasing the transcriptional expression of the Scr homology-2 domain that contains phosphatase-1 (SHP-1), leading to the dephosphorylation of platelet-derived growth factor receptor-β (PDGFR-β), critical for retinal pericyte survival. This inactivation of PDGFR-β results in pericyte loss, which results in the formation of microaneurysms and therefore the attraction of leukocytes, which are early histopathological signs of DR [43].

The most commonly used current treatments for DR are mainly directed towards the more advanced phases of the disease, when PDR or DME have already developed. These treatments include laser photocoagulation, intravitreous injection of compounds with anti-VEGF action or vitreoretinal surgery [44]. Although in many cases they slow the progression of DR and present some effectiveness in preventing vision loss, they are not effective in all patients and are invasive and destructive therapies, so new, less aggressive therapies that are more effective in the early stages of DR are needed.

## 3. The Importance of Nutraceuticals

Despite numerous studies that have sought to identify possible drugs for the prevention and treatment of DR, little attention has been given to nutraceuticals. Nutraceuticals are natural functional foods that promote various health benefits, which include a wide variety of compounds such as vitamins, antioxidants, minerals, fatty acids and amino acids that can prevent or delay the progression of certain diseases. Various studies have shown that nutraceuticals promote multiple therapeutic benefits and provide protection against various diseases. In diabetes, the use of nutraceuticals contributes to improve insulin sensitivity, metabolism regulation and lower hyperglycemia [45]. Molecules such as flavonoids and carotenoids have been proven to have significant antioxidant and anti-inflammatory effects [46]. In many animal models and humans studies, it has been shown that flavonoids, a large family of compounds that are extracted from plants, can prevent or attenuate complications associated with DR as they can modulate lipid and carbohydrate metabolism and insulin resistance, mitigate hyperglycemia and suppress oxidative stress and inflammatory processes [47].

Red fruits are among the most sought-after by consumers with health concerns. These fruits, such as raspberry (*Rubus idaeus*), blueberry (*Vaccinium corymbosum*), strawberry (*Fragaria ananassa*), blackberry (*Rubus ulmifolius*) and cranberry (*Vaccinium macrocarpon*), are rich in dietary fiber and organic acids, low in fat and calories and contain high amounts of antioxidant molecules, such as polyphenols, that prevent the fruit from oxidation against environmental factors, such as light, oxygen and microbiological contamination [48]. Polyphenols have various important biological properties such as anti-viral, anti-bacterial, anti-inflammatory, anti-cancer and, most importantly, antioxidant, which promote the capacity of radical scavenging that is based on the ability to turn free radicals into more stable ones, or by reducing the production of ROS caused by intracellular mechanisms in the mitochondria [49]. This capability is the main antioxidant defense system as intracellular antioxidants neutralize the damaging effect of free radicals preserving the cellular redox homeostasis. Due to these effects, polyphenols have also been explored to create cosmetics, creams, nutraceuticals and dietary supplements which also have proven benefits to human health.

## 4. Diabetic Retinopathy and the Benefits of Flavonoids

**Figure 1:**
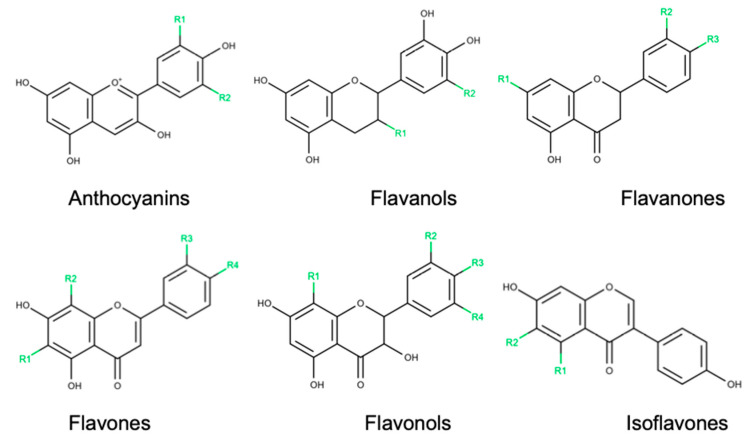
Chemical structures of flavonoid subclasses.

Polyphenols, chemically characterized as compounds with phenolic structural features, constitute one of the most numerous and widely distributed groups of natural products in the plant kingdom. This group of natural products is highly diverse and contains several sub-groups of phenolic compounds. Flavonoids, one polyphenol sub-group that accounts for about 60% of all polyphenols [50], can be found in fruits and vegetables with specific biological characteristics that include anti-inflammatory, antiviral and antioxidant effects. Flavonoids can be categorized in six classes according to their chemical structure, namely anthocyanins, flavanols, flavanones, flavones, flavonols and isoflavones [51] (Figure 1).

Flavonoids can modulate carbohydrate and lipid metabolism, improve insulin resistance, attenuate hyperglycemia, improve β-cell function and improve the management of inflammatory processes, which could help to prevent the development of long-term chronic diabetic complications, such as diabetic retinopathy [52].

### 4.1. Anthocyanins

Anthocyanins are natural pigments that provide blue, red and purple colors in flowers, fruits and other plant structures [53]. This flavonoid subclass is present in various fruits, being in high concentrations in berries and cherries [54]. Besides being used as colorants, anthocyanins have various beneficial effects on DR (Table 1).

A study performed in human retinal capillary endothelial cells (HRCECs) showed that blueberry anthocyanin extract (BAE) and its predominant constituents, malvidin (Mv), malvidin-3-glucoside (Mv-3-glc) and malvidin-3-galactoside (Mv-3-gal), prevented the high glucose-induced injury in HRCEs. It was observed that a reduction of the inflammatory and oxidative environment, caused by a decrease in pro-oxidant enzymes endothelial nitric oxide synthase (eNOS) and NADPH oxidase 4 (Nox4), increased antioxidant enzymes catalase (CAT) and superoxide dismutase (SOD), and inhibition of the intercellular adhesion molecule-1 (ICAM-1) and NF-κB pathway. Additionally, angiogenesis was compromised by a decrease in the VEGF level and inhibition of the protein kinase B (Akt) pathway [55]. The protective effects of BAE were also evaluated in vivo. The anthocyanins were administered orally in streptozotocin (STZ)-induced T1DM rats. As observed in vitro, ROS levels lowered, antioxidant enzymes glutathione (GSH) and glutathione peroxidase (GPx) increased, and VEGF levels diminished. Additionally, malondialdehyde (MDA) and IL-1β levels decreased. These results show that BAE has anti-inflammatory and antioxidant effects. Since BAE augmented nuclear factor erythroid 2-related factor-2 (Nrf2) and HO-1 mRNA levels and the nuclear location of Nrf2 and HO-1 protein levels, it was suggested that the oxidative stress and inflammation could be regulated by the Nrf2/HO-1 pathway [56].

Kim and colleagues showed that bilberries can prevent or delay the onset of DR by preventing BRB disruption [57]. STZ-induced T1DM rats, treated with *Vaccinium myrtillus* extract (VME) that contains 15 different anthocyanins, exhibit less VEGF expression and more tight junction proteins (zonula occludens-1, occludin and claudin-5) in the retina. Furthermore, treated rats had less fluorescein leakage. These results show that BRB breakdown was inhibited by VME [57].

### 4.2. Flavanols

Flavanols, also referred to as flavan-3-ols or catechins, are present in high concentrations in cocoa, grapes, tea [58] and red wine [59].

Advanced glycation end products (AGEs) are direct contributors in the initiation and progression of diabetic retinopathy [60,61]. (−)—Epicatechin may improve retinal vascular cell injuries by reducing the AGEs burden in vitro and in vivo. Glycated human serum albumin isolated from diabetic patients incubated with (−)—epicatechin presented higher AGE breaking activity. This was also verified in normoglycemic rats injected with AGE-modified rat serum albumin. Additionally, the AGE burden lowered, as well as vascular apoptosis [62]. Skopinski and colleagues showed that plant-derived flavanols (epigallocatechin (EGC) and epigallocatechin gallate (EGCG)) inhibited the angiogenic effects of sera obtained from type 2 diabetic patients with non-proliferative retinopathy in Balb/c mice [63].

Green tea (*Camellia sinensis*) is characterized by its high flavonoid content (20–30% of the dry weight) [64]. Green tea exerts protective effects against glutamate toxicity in the diabetic retina. Silva and colleagues have shown that oral administration of green tea reduced some retinal complications of diabetes in two animal models. The increase in the expression of glial fibrillary acidic protein (GFAP), oxidative retinal markers and glutamine synthetase levels was prevented by green tea. In addition, the decrease in occludin, NMDAr1 subunit and GLAST-1 (GLutamate ASpartate Transporter 1) verified in diabetic animals was also reduced in green tea-treated animals. Diabetic spontaneously hypertensive rats (SHR) also exhibit BRB breakdown and impaired electroretinography recordings. Müller cells exposed to high-glucose medium produced higher levels of ROS and glutamine synthetase, but reduced levels of GSH, glutamate transporter and glutamate receptor [65]. Similarly, ARPE-19 cells exhibited increased ROS production accompanied by decreased expression of claudin-1 and glutamate transporter. Treatment with green tea fully restored all the above-mentioned alterations in diabetic animals as well as in retinal cells [65]. Additionally, green tea also decreased superoxide production, acellular capillaries and pericyte ghosts in vivo [66]. Metalloproteinase-9 (MMP-9) promotes neovascularization and vascular permeability present in late DR. EGCG, green tea’s most active compound, inhibited 12-O-tetradecanoylphorbol-13-acetate (TPA)-induced MMP-9 and TNF-α mRNA and protein expression levels in human retinal pigment epithelial cells (HRPECs). EGCG exerted antiapoptotic effects by decreasing ROS levels and attenuating mRNA expression of MMP-9, VEGF and VEGF Receptor-2 in ARPE-19 cells [67]. Furthermore, HRPECs exposed to VEGF and EGCG presented less proliferation, vascular permeability and tube formation. In vivo, EGCG reduced VEGF-induced vascular leakage and permeability [67].

STZ-induced T1DM rats treated with catechin presented diminished pro-inflammatory cytokines levels (IL-1β, IL-6 and TNF-α) as well as downregulated NF-κB. These results show that catechin exerts anti-inflammatory effects [68]. The effects of flavanols on DR are summarized in Table 2.

### 4.3. Flavanones

Flavanones are abundant in fruits and fruit juices of the *Citrus* genus such as oranges, bergamots, lemons and grapefruit [69].

Naringenin presents antioxidant, neuroprotective and anti-apoptotic effects in the diabetic retina. It ameliorated the oxidative stress by lowering GSH levels and thiobarbituric acid reactive substances (TBARs) in STZ-induced T1DM rats. Furthermore, brain-derived neurotrophic factor (BDNF), tropomyosin related kinase B (TrkB) and synaptophysin were augmented, preventing neurodegeneration. Naringenin also improved the level of apoptosis-regulatory enzymes by decreasing Bax and caspase-3 and increasing Bcl-2 levels [70].

Eriodictyol exerts antioxidant, anti-inflammatory and antiapoptotic effects via the Nrf2/HO-1 pathway. This flavanone attenuated retinal inflammation by diminishing TNF-α, ICAM-1, VEGF and eNOS levels in STZ-induced T1DM rats. By decreasing these factors, eriodictyol prevented BRB breakdown. Moreover, eriodictyol inhibited plasma lipid peroxidation, a feature induced by ROS [71,72]. In vitro, eriodictyol also ameliorated the oxidative stress by lowering ROS levels and augmenting SOD, GPx and CAT activity. Additionally, eriodictyol enhanced cell viability, heme-oxygenase-1 expression and Nrf2 nuclear translocation, an important regulator of oxidative stress [73].

Hesperitin presents vasoprotective and antioxidant effects in the diabetic retina. PKC-β is an important mediator in the VEGF pathway, being involved in vascular tissues anomalies. This flavanone diminished PKC-β and VEGF expression in STZ-induced T1DM rats. Hesperitin also reduced vascular permeability, leakage and dilation of the vessels, and reduced vascular basement membrane (BM) thickness [74]. Additionally, hesperitin restored GSH levels, SOD and CAT activities. Pro-inflammatory cytokines levels (TNF-α and IL-1β) decreased, as well as caspase-3 and GFAP expression. Aquaporin-4 (AQP4) is highly expressed in Müller cells and astrocytes, being associated with neuronal and glial swelling when overexpressed. This overexpression observed in diabetic retinas was reduced by hesperitin [75]. Photoreceptors are responsible for converting light stimuli into electrical stimuli for visual processing. Accordingly, if there is degeneration of these sensory neurons, it will lead to visual loss [76]. Light and transmission electron microscopic studies showed that hesperitin reduced photoreceptors cell death. It was also observed that edematous Müller cells’ feet and BM thickness were diminished [75]. Hesperidin suppresses BRB injuries and prevents the reduction in retina thickness induced by diabetes in an STZ-induced T1DM rat model. Hyperglycemia is involved in the development of DR via increasing aldose reductase activity. Hesperidin reduced blood glucose levels, aldose reductase activity, ICAM-1, IL-1β, TNF-α, VEGF and AGEs. Additionally, MDA levels were significantly reduced, and SOD activity increased, improving the oxidative state [77]. This was also observed in vitro by the downregulation of ROS, MDA and protein carbonyl levels, and the increase in SOD, CAT, GPx and GSH levels. High glucose-exposed retinal ganglion cell 5 (RGC-5) and ARPE-19 cells treated with hesperidin had less cell loss and restored mitochondrial function, including an increase in the mitochondrial membrane potential and inhibition of cytochrome c release, preventing programmed cell death. Additionally, this flavanone inhibited cell apoptosis via downregulating caspase-9, caspase-3, caspase-2 and the Bax/Bcl-2 ratio [78,79]. Lastly, the phosphorylation of c-Jun N-terminal kinase (JNK) and p38 MAPK was diminished, protecting retinal cells against ROS injury and cellular death [78]. The effects of flavanones on DR are summarized in Table 3.

### 4.4. Flavones

Flavones are extensively distributed in cereal grains, reported to also be in herbs [80].

12/15-lipoxygenase is linked to the development of microvascular dysfunction during DR. Othman and colleagues suggested that baicalein had anti-inflammatory, antioxidant and anti-hyperpermeability effects in diabetic mouse retinas, acting as a 12/15-lipoxygenase (12/15-LOX) inhibitor. Mice treated with baicalein had reduced ICAM1, VCAM-1, IL-18, TNF-α, IL-1β and IL-6 expression levels, reduced GFAP and VEGF expression in Müller cells and diminished vascular abnormality and ganglion cells loss in the retina [81,82]. The treatment with this flavone also prevented the increase and the activation of microglia, restored zonula occludens-1 (ZO-1) and protein tyrosine phosphatase pSHP1 levels [81,82]. Protein tyrosine phosphatase decrease has a role in maintaining endothelial barrier function, potentially by inhibiting the VEGF/VEGF-R2 pathway. Additionally, baicalein reduced pVEGF-R2 levels and significantly decreased 12- and 15-hydroxyeicosatetreanoic acids, ROS generation and NADPH oxidase 2 (NOX2) expression in diabetic retina [82,83].

Baicalin reduced cell death and apoptosis, inhibited the release of IL-1β, IL-6 and IL-8 and diminished ROS levels, ameliorating the inflammatory and oxidative states of ARPE-19 and human retinal microvascular endothelial cells (HRMEC) exposed to high glucose [84]. MicroRNA (miRNAs) constitute a class of small non-coding RNA that regulate the expression of genes at the post-transcriptional level by binding to target sites directly or promoting mRNA degradation [85]. miR-145 is one of various miRNAs that are involved in DR, and several works have shown that miR-145 levels are altered in diabetic retinas and in cellular models of DR [84,86,87]. Treatment with baicalin increased the levels of miR-145 and consequently inhibited the NF-κB and p38MAPK pathways [84], which are linked to a greater permeability of the blood vessels, one of the abnormalities observed in DR [88].

Mei and colleagues showed that scutellarin alleviates BRB breakdown in animal and cellular models of DR. This flavone diminished NF-κB and TNF-α expression in the BV-2 cell line exposed to high glucose. TNF-α-exposed HRECs and ARPE-19 cells treated with scutellarin augmented claudin-1 and claudin-19 expression, ROS formation and Nrf2 nuclear accumulation. These results were also observed in STZ-induced T1DM rats. Both in vivo and in vitro, this flavone reduced microglia cell activation and phosphorylation of ERK1/2 [89]. Scutellarin also decreased cell viability and VEGF levels via reducing hypoxia-inducible factor 1 (HIF-1α) mRNA and protein levels in HRECs exposed to high glucose and hypoxia-mimetic agent. Additionally, scutellarin diminished the activity of NADPH oxidase, the principal source of ROS [90].

Nepetin decreased production and expression of IL-6, IL-8 and MCP-1 in IL-1β-treated ARPE-19 cells. The nuclear translocation of NF-κB p65 subunit was lowered via suppressing the phosphorylation of inhibitor of nuclear factor kappa B (IκB) and IκB kinase (IKK). Moreover, nepetin decreased the phosphorylation of ERK1/2, JNK and p38 MAPK induced by IL-1β. These results show that nepetin ameliorated the inflammatory responses via suppressing the NF-κB and MAPKs pathways [91].

Silybin diminishes obliterated retinal capillaries, a hallmark of early morphological pathology in DR. Additionally, silybin significantly reduced retinal vascular leukocyte adhesion (leukostasis) and the ICAM-1 level in STZ and high-fat diet-induced T2DM rats [92].

Chrysin exerts protective effects in diabetes-associated visual cycle impairment. Chrysin ameliorated glucose-induced neovascularization by diminishing VEGF and IGF-1 levels and restoring pigment epithelium-derived factor (PEDF) levels in RPE cells [93]. Chrysin-treated db/db mice restored the ONL thickness and augmented visual cycle-related enzymes levels (RPE65, lecithin retinol acyltransferase (LRAT), retinol dehydrogenase 5 (RDH5), rhodopsin, receptor of the cellular retinol-binding protein, cellular retinaldehyde-binding protein-1, interphotoreceptor retinoid-binding protein and retinoic acid 6) [93]. Chrysin decreased AGE production and RAGE induction in glucose-treated RPE cells and increased PEDF, RPE65, LRAT and RDH5 in AGE-BSA-exposed RPE cells. In glucose-exposed RPE cells and mice, chrysin inhibited endoplasmic reticulum (ER) stress sensor proteins IRE1α and ATF6, preventing ER stress-mediated loss of visual cycle proteins via the AGE/RAGE pathway [93].

Diosmin, a flavone glycoside (3′,5,7-trihydroxy-40-methoxyflavone-7-rhamnoglucoside) found in citrus fruits, is a venoactive and a vascular protector [94]. It is the main component of Daflon, a dietary nutraceutical, and is used in the treatment of symptoms and signs related to venous insufficiency (heavy legs, pain, tiredness, edema) and in the symptomatic treatment of hemorrhoidal crisis [95]. The administration of diosmin in an animal model of ischemic/reperfusion showed protective effects in the BRB, avoiding the increase in its permeability, reducing the levels of VEGF and relieving edema [96]. It was also observed that diosmin also exerted a protective effect in the neural component of the retina, preventing the reduction in the total thickness of the retina, avoiding the reduction in the number of ganglion cells and the changes in the a- and b-waves of the electroretinograms induced by the I/R. In this same model, it was also observed that the administration of diosmin reduced the levels of MDA and increased the activity of the antioxidant enzymes SOD, CAT and GSH-Px in the retina [97]. Similar results were observed in ARPE-19 cells exposed to high glucose. In this cell line, diosmin increased cell viability, through the decrease in apoptosis, and ameliorated the decrease in SOD and GSH-Px enzymatic activities, with a consequent reduction in the ROS levels [98]. Table 4 summarizes the effects of flavones on RD.

### 4.5. Flavonols

Flavonols are usually present in a variety of vegetables, fruits, tea and wine [99].

Under high-glucose conditions, HRECs presented elevated VEGF and placenta growth factor (PGF) mRNA and protein levels. Kaempferol incubation suppressed the increase in both angiogenic factors, therefore inhibiting angiogenesis. Moreover, kaempferol inhibited high glucose-induced expression of PI3K and the phosphorylation of specific kinases (Src, Akt1 and Erk1/2), suggesting that this flavonol exerts the anti-angiogenic effects by inhibition of the Src-Akt1-Erk1/2 signaling pathway [100]. α-Glucosidase and α-amylase are enzymes in the digestive system that hydrolyze dietary carbohydrates and produce absorbable glucose. The degradation of dietary starch leads to postprandial hyperglycemia in patients with diabetes. Kaempferol inhibits these key enzymes and, through this mechanism, can reduce and control postprandial blood glucose spike, which is an effective approach to alleviate and treat T2DM [101].

Quercetin has a protective action against injuries caused by diabetes in the retina [102,103,104] (Table 5). Quercetin attenuated high glucose-induced apoptosis and inflammation by lowering ROS levels, pro-inflammatory molecules, MCP-1 and IL-6. It has, recently, been suggested that miR-29b may be beneficial in the treatment of DR, as it has antiangiogenic effects on diabetic retinas through inhibition of retinal microvascular endothelial cells proliferation, migration and angiogenesis [105]. miR-29b expression was higher in ARPE-19-treated cells, being noticed that quercetin protective effects were lower when miR-29b was suppressed. Moreover, PTEN/Akt pathway activation was promoted and the NF-κB pathway was inhibited via a miR-29b-dependent way [104]. In vivo, quercetin also inhibited NF-κB expression and caspase-3, presenting antiapoptotic effects [103]. This flavonol downregulates proteins that play an important role in neovascularization, namely MMP-9 and VEGF [102]. Quercetin augmented antioxidant enzymes (GSH, SOD and CAT) and diminished pro-inflammatory cytokines (TNF-α and IL-1β). Additionally, quercetin lowered GFAP and AQP4 expression [103].

Rutin exerts antiapoptotic effects via decreasing caspase-3 and increasing the expression of both neurotrophic factors (BDNF and nerve growth factor (NGF)) and Bcl-2 levels in the retina of T1DM rats [106]. This flavonol lowered VEGF, TNF-α and aldose reductase protein levels and prevented vascular leakage of fluorescein in the retina of diabetic animals. Furthermore, it also increased the total antioxidant capacity of the retinas [107].

Galangin alleviates BRB breakdown via inhibiting microglia-mediated inflammation and further reversing TNF-α-induced BRB dysfunction via activating the Nrf2 pathway [108]. HRECs and ARPE-19 cells exposed to TNF-α and treated with galangin increased tight junction enzymes expression (claudin 1 and occludin) and diminished BRB injury. Galangin also induced the activation of Nrf2, which regulates several antioxidant genes [108]. Both in vivo and in vitro, galangin diminished microglial cells activation, ROS formation, phosphorylation of ERK1/2, NF-κB and early growth (Egr1) protein and expression of TNF-α. Moreover, galangin attenuation on BRB breakdown was lowered in Nrf2 knockout T1DM mice [108].

Exposure of bovine retinal pericytes (BRP) to AGE of bovine serum albumin (AGE-BSA) induced BRP migration, which led to pericyte loss, and stimulated ERK1/2 phosphorylation. AGE-BSA also promoted cytoskeletal proteins (FAK and paxillin) phosphorylation, supporting cell motility [109]. Similar results were observed when AGE-BSA was intravitreally injected in rats [109]. Myricetin suppressed pericytes migration and lowered ERK1/2-FAK-1-paxillin phosphorylation both in vitro and in vivo [109]. ARPE-19 cells exposed to glucose oxidase generated H_2_O_2_-induced oxidative stress. Myricetin derivatives isolated from *Syzygium malaccense* ameliorated the oxidative environment by diminishing intracellular ROS via activation of Nrf2 and superoxide dismutase (SOD2), along with downregulating the transcription of nitric oxide producer (iNOS) [110]. High glucose-treated ARPE-19 cells exposed to myricetin derivatives attenuated the high glucose-induced stress condition by lowering ROS levels, and inhibited AGE products. Myricetin derivatives also upregulated antioxidant proteins and other protective factors. Additionally, the protective effects of this flavonol against the formation of AGE is correlated with its capacity of downregulating the expression of inflammatory factor NFkB1 and RAGE [111].

Icariin, orally administered to STZ-induced T1DM rats, prevented the decrease in the expression of rat endothelial cell antigen (RECA), Thy-1, Brn3a and collagen IV observed in diabetic retinas [112]. Xi and colleagues also observed that icariin treatment prevented the decrease in the number of Müller cells, immunostained for carbonic anhydrase II. Additionally, icariin enhanced neurite growth in cultured RGCs isolated from diabetic and normal rats. However, in the same work, a reduction in VEGF levels in the retina of diabetic animals was found, contrary to what is normally described. The administration of icariin not only prevented the reduction in the angiogenic factor induced by diabetes but also increased the VEGF levels in the control animals, thus questioning the use of this flavonol as a protective compound against DR [112].

### 4.6. Isoflavones

Isoflavones, one of the most estrogenic compounds, present in soybeans, soybeans products and other legumes [113] also exert beneficial effects on RD (Table 6).

Biochanin A administration in STZ-induced T1DM rats lowered blood glucose levels and decreased angiogenesis and inflammation via suppressing VEGF, TNF-α and IL-1β [114].

Jia and colleagues demonstrated that formononetin reduces H_2_O_2_-induced apoptosis and activation of the NF-κB pathway in RGC-5 cells. Treated cells showed less oxidant stress by decreasing superoxide anions, MDA and 8-hydroxy-2-deoxyguanosine (8-OHdG) levels and increasing MnSod activity. Moreover, NF-κB was downregulated, contributing to lower the inflammation state [115].

Puerarin exerts antiapoptotic and antioxidant effects in vitro and in vivo. STZ-induced T1DM rats treated with puerarin showed a reduction in morphological changes in the inner nuclear layer (INL) and outer nuclear layer (ONL), and augmented ONL thickness and b-wave amplitude [116,117,118]. The oxidative state was attenuated by a decrease in AGE accumulation and MDA content, and an increase in SOD activity [117,118,119]. Puerarin also downregulated VEGF, RAGE and HIF-1α expression and lowered STAT3 expression and protein levels in the retina [116,117,118]. Treated T1DM rats showed less retinal cell apoptosis and inhibited NF-κB p65 activity [120]. This isoflavone relieved RPE cells apoptosis in diabetic rats via reducing nitrotyrosine (NT), complement 3, iNOS mRNA and Fas/Fasl protein expressions [121,122]. AGE-BSA-induced apoptosis in bovine retinal pericytes was attenuated, as well as ROS, NF-κB and NADPH oxidase activity through inhibition of the phosphorylation of p47phox and Rac1 subunits. Apoptosis was also decreased in rat retinal pericytes [123]. Rat retinal capillary endothelial cells (TR-iBRB2) exposed to IL-1β and puerarin showed less leukostasis and cell apoptosis [124]. NMDA-induced injuries in RGCs were attenuated by puerarin. Puerarin diminished NO levels and reduced iNOS and neuronal NOS (nNOS) expression. Oxidant enzymes were also reduced (ROS and MDA) and SOD was augmented. NMDA-induced apoptosis was attenuated by increasing Bcl-2 expression and lowering Bax expression and caspase-3 activity. Additionally, puerarin inhibited JNK and p38 phosphorylation. In vivo, puerarin also prevented RGC loss [125].

Kumar and colleagues demonstrated that isoflavones extracted from *Caesalpinia pulcherrima* (3,6,7,40,50-pentamethoxy-5,30-dihydroxyflavone) can inhibit aldose reductase, delaying the DR onset. STZ-induced diabetic rats had decreased antioxidant enzyme activity (SOD, CAT and GPx) and GSH levels, being ameliorated by 3,6,7,40,50-pentamethoxy-5,30-dihydroxyflavone. Additionally, TBARs and protein carbonyl levels were significantly decreased [126].

## 5. Clinical Studies

Very few studies have been conducted to understand how nutraceuticals can improve diabetes and DR. A clinical trial with 10,054 participants, of which 546 were addressed to measure the risk of developing diabetes, demonstrated that higher intake of quercetin and myricetin for one year decreases the risk of developing T2DM [127]. A meta-analysis of randomized controlled clinical trials evaluated the impact that green tea catechins, with or without caffeine, can have on glycemic control markers in 1584 subjects. Results showed that the administration of these substances could significantly reduce fasting blood glucose, although there were no verified significant differences in fasting blood insulin, glycated hemoglobin (HbA1c) and homeostatic model assessment of insulin resistance (HOMA-IR) [128]. A randomized controlled trial assessed the effects of an oral combination of flavonoids, *Centella asiatica* and *Melilotus* for the treatment of diabetic cystoid macular edema without macular thickness in 70 type 2 diabetic patients. Results demonstrated that retinal sensitivity was preserved in treated patients when compared to the untreated group, although no significant differences in visual acuity, stability fixation, mean central retinal thickness, HbA1c percentage, microalbuminuria and blood pressure were observed [129]. Another study, which evaluated the effects of purified anthocyanins in 160 patients for 12 weeks, exhibited that the supplementation of anthocyanins promoted an increase in serum adiponectin and a decrease in fasting glucose in newly diagnosed diabetics [130]. Mahoney et al. conducted a study that used information of 381 diabetic people from the National Health and Nutrition Examination Survey (NHANES) between 2003 and 2006 to evaluate if a flavonoid-rich diet impacts DR and diabetes-related biomarkers. The results demonstrated that participants with a high intake of flavonoids diet lowered the risk of developing DR by 30%. Additionally, they also presented lower C-reactive protein, HbA1C and glucose levels [131]. A clinic-based case–control study revealed that people who regularly drink green tea for at least a year presented a 50% lower risk of developing DR than those who do not drink green tea [132]. A multi-center field study that assessed Pycnogenol^®^’s effect on the progression of visual acuity in patients with T1DM and T2DM who had DR, in which 1169 people were treated with Pycnogenol^®^ for six months, demonstrated that Pycnogenol^®^ prevented the progression of visual loss. However, there were no significant improvements in patients’ sight [133,134]. Another study demonstrated that people treated with antioxidant supplementation containing Pycnogenol^®^ for six months presented lower ROS levels and central macular thickness [135]. Additionally, Steigerwalt et al. evaluated the effects of Pycnogenol^®^ in the early stages of DR. Results exhibited that people treated with Pycnogenol^®^ for two months presented visual and baseline improvement [134].

Although some of these clinical trials show positive effects in diabetic patients, difficulties arise in analyzing the clinical trial’s results since they are variable and, in some cases, even controversial. Many of these studies focus on T2DM patient populations and some clinical trials deal with patients that are in different stages of the disease, making it more difficult to correlate with the accuracy of the results [136]. A summary of clinical studies related to flavonoids and DR is listed below (Table 7).

## 6. Conclusions

Flavonoids can provide an effective and safe alternative to conventional drugs and therapies that are used to prevent and treat DR, one of the major complications of diabetes. As shown by several studies performed in vivo, using animal models of diabetes, and in vitro, using different cell cultures, flavonoids can prevent the disruption of the BRB, decrease the release of proinflammatory mediators, improve the oxidative state and prevent the reduction in retina thickness by attenuating apoptosis and neurodegeneration. These may contribute to the beneficial effects of the consumption of flavonoids observed in clinical studies. Despite the small number, these studies have shown that consumption of flavonoids, in the diet or through supplements, exerts beneficial effects at several stages: preventing the onset of diabetes, the development of DR in diabetics and, in diabetics with DR, flavonoids prevent the worsening of DR. However, nutraceuticals should be used as supplements to a healthy and balanced diet and not as a magic bullet that in itself prevents all the complications of diabetes. In conclusion, the data presented in this review strongly suggest that dietary supplementation with flavonoids or with flavonoids-rich nutraceuticals may be an effective, economical and safe way to prevent or limit the progression of DR and the concomitant visual impairments, thus improving the quality of life of millions of diabetics.

## Figures and Tables

**Table 1:** Effects of anthocyanins on cellular and molecular mechanisms affected by diabetic retinopathy.

Name of Substances/Compound	Dose	Model	Observations	Reference
Blueberry anthocyanins	20, 40 or 80 mg/kg orally administered for 12 weeks	STZ-induced T1DM rats	↑ antioxidant capacity of the retina	[56]
			↑ GSH and GPx activity	
			↑ Nrf2 and HO-1 mRNA and protein levels	
			↓ weight loss and blood glucose levels	
			↓ VEGF level and ROS levels	
			↓ MDA and IL-1β levels	
	10 μg/mL of BAE, Mv, Mv-3-glc or Mv-3-gal for 24 h	High glucose-exposed HRCECs	↑ CAT and SOD, ICAM-1 and NF-κB pathway	[55]
			↑ cell viability	
			↓ VEGF levels and Akt pathway	
			↓ ROS, eNOS and NO levels	
			↓ Nox4 expression	
*Vaccinium myrtillus* extract	100 mg/kg orally administered for 6 weeks	STZ-induced T1DM rats	↑ zonula occludens-1, occludin and claudin-5 levels	[57]
			↓ fluorescein leakage	
			↓ VEGF levels	

Akt: protein kinase B, BAE: blueberry anthocyanin extract, CAT: catalase, eNOS: endothelial nitric oxide synthase, GPx: glutathione peroxidase, GSH: glutathione, HO-1: heme oxygenase 1, HRCECs: human retinal capillary endothelial cells, ICAM-1: intercellular adhesion molecule 1, IL-1β: interleukin-1β, MDA: malondialdehyde, Mv: malvidin, Mv-3-gal: malvidin-3-galactoside, Mv-3-glc: malvidin-3-glucoside, NF-κB: nuclear factor kappa-B, NO: nitric oxide, Nrf2: nuclear factor erythroid 2-related factor-2, Nox4: NADPH oxidase 4, ROS: reactive oxygen species, SOD: superoxide dismutase, STZ: streptozotocin, T1DM: type 1 diabetes mellitus, VEGF: vascular endothelial growth factor, ↑: increase, ↓ decrease.

**Table 2:** Effects of flavanols on cellular and molecular mechanisms affected by diabetic retinopathy.

Name of Substances/Compound	Dose	Model	Observations	Reference
(−)—Epicatechin	50 and 100 mg/kg for 2 weeks	Unmodified rat serum albumin and AGE-modified rat serum albumin intravenous injected (normoglycemic) rats	↑ AGE breaking activity	[62]
			↓ AGE burden and vascular apoptosis	
	10, 25, 50, 100, 250, 500 and 1000 μg/mL	Collagen–glycated bovine serum albumin complexes and glycated human serum albumin	↑ AGE breaking activity	
Epigallocatechin	2, 20 and 200 µg	Balb/c mice intradermally injected with NPDR patients serum	↓ angiogenic effects	[63]
Green tea	5.7 g/kg for 12 weeks	STZ-induced T1DM rats	↓ occludin, NMDAr1 subunit and GLAST levels	[65]
			↓ BRB breakdown	
			↓ impaired electroretinography recordings	
			↓ GFAP, oxidative retinal markers and glutamine synthetase levels	
	1, 10 or 100 µg/mL	High glucose-exposed PRRMC, rMC-1 and ARPE-19 cells	↑ GSH, GLAST and NMDAR1 protein levels	
			↓ ROS and glutamine synthetase	
			↓ claudin-1 levels	
	100 mL for 12 months	STZ-induced T1DM rats	↓ superoxide production, acellular capillaries and pericyte ghosts	[66]
Epigallocatechin gallate	2, 20 and 200 µg/mL	Balb/c mice intradermally injected with NPDR patients serum	↓ angiogenic effects	[63]
	200 mg/kg orally administered for 4 days	VEGF intradermally injected Balb/c mice and Sprague-Dawley rats	↓ vascular leakage and permeability by BRB breakdown	[67]
	1, 10, 25 and 50 μM for 1.5, 24 or 72 h	TPA and TNF-α-exposed HRPECs and ARPE-19 cells	↓ MMP-9 levels	
			↓ cell death and MMP-9, VEGF and VEGF Receptor-2 mRNA expression	
		H_2_O_2_-exposed ARPE-19 cells		
			↓ ROS levels	
		VEGF-exposed HRMECs	↓ cell proliferation, vascular permeability and tube formation	
Catechin	50, 100 and 200 mg/kg by intravitreal injection for 8 weeks	STZ-induced diabetic rats	↑ HSP27 levels	[68]
			↓ IL-1β, IL-6 and TNF-α levels	
			↓ NF-κB pathway	

AGE: advanced glycation end products, ARPE-19: human retinal pigment epithelial cell line, Balb/c: Bagg’s albino C mice substrain, BRB: blood–retinal barrier, GFAP: glial fibrillary acidic protein, GLAST: glutamate aspartate transporter 1, GSH: glutathione, H_2_O_2_: hydrogen peroxide, HRMECs: human retinal microvascular endothelial cells, HRPECs: human retinal pigment epithelial cells, HSP27: heat shock protein 27, IL-1β: interleukin-1β, IL-6: interleukin-6, MMP-9: metalloproteinase-9, NF-κB: nuclear factor kappa-B, NMDAr1: NR1 subunit of N-methyl-D-aspartate receptor, NPDR: non-proliferative diabetic retinopathy, PRRMC: primary rat retina Müller cells, rMC-1: transformed rat retinal Müller cell line, ROS: reactive oxygen species, STZ: streptozotocin, T1DM: type 1 diabetes mellitus, TNF-α: tumor necrosis factor alfa, TPA: 12-O-tetradecanoylphorbol-13-acetate, VEGF: vascular endothelial growth factor, ↑: increase, ↓ decrease.

**Table 3:** Effects of flavanones on cellular and molecular mechanisms affected by diabetic retinopathy.

Name of Substances/Compound	Dose	Model	Observations	Reference
Naringenin	50 mg/kg orally administered for 5 weeks	STZ-induced T1DM rats	↑ GSH levels	[70]
			↑ BDNF, TrkB and synaptophysin levels	
			↑ Bcl-2 levels	
			↓ TBARs levels	
			↓ Bax and caspase-3 levels	
Eriodictyol	0.1, 1 and 10 mg/kg orally administered for 10 days	STZ-induced T1DM rats	↓ TNF-α, ICAM-1, VEGF and eNOS levels	[72]
			↓ plasma lipid peroxidation	
			↓ BRB breakdown	
	5, 10 and 20 μM for 24 h	High glucose-exposed RGC-5 cells	↑ SOD, GPx and CAT activity	[73]
			↑ cell viability and heme-oxygenase-1 expression	
			↑ Nrf2 nuclear translocation	
			↓ ROS levels	
			↓ TNF-α and IL-8 levels	
			↓ cell apoptosis	
Hesperetin	200 mg/kg orally administered for 24 weeks	STZ-induced T1DM rats	↓ VEGF and PKC-β expression	[74]
			↓ vascular permeability and leakage	
			↓ dilated vessels	
			↓ basement membrane thickness	
	100 mg/kg orally administered for 24 weeks	STZ-induced T1DM rats	↑ GSH levels	[75]
			↓ TNF-α and IL-1β levels	
			↓ caspase-3, GFAP and aquaporin-4 expression	
			↓ photoreceptors cell death	
			↓ edematous Müller cells’ feet, degenerated photoreceptor layer and basement membrane thickness	
Hesperidin	100 and 200 mg/kg intragastrically administered for 12 weeks	STZ-induced T1DM rats	↑ retina thickness	[77]
			↑ SOD activity	
			↓ blood glucose, aldose reductase activity, ICAM-1, IL-1β, TNF-α, VEGF and AGEs levels	
			↓ BRB breakdown	
			↓ MDA levels	
	12.5, 25 and 50 µmol/L for 48 h	High glucose-exposed RGC-5 cells	↑ SOD, CAT, glutathione peroxidase activities and GSH levels	[78]
			↓ cell loss and cytochrome c release	
			Reverted ∆Ψm loss	
			↓ ROS, MDA and protein carbonyl levels	
			↓ caspase-9, caspase-3 and Bax/Bcl-2 levels	
			↓ c-Jun N-terminal kinase phosphorylation and p38 MAPK	
	10, 20 or 40 µM for 48 h	High glucose-exposed ARPE-19 cells	↑ cell viability	[79]
			↑ glutathione peroxidase, SOD and CAT activities and GSH levels	
			↓ ROS production	
			↓ caspase-9/3, cytochrome c release and Bax/Bcl-2 ratio	

∆Ψm: mitochondrial membrane potential, AGE: advanced glycation end products, ARPE-19: human retinal pigment epithelial cell line, Bax: Bcl-2 associated X protein, Bcl-2: B cell lymphoma-2 protein BDNF: brain-derived neurotrophic factor, BRB: blood–retinal barrier, CAT: catalase, eNOS: endothelial nitric oxide synthase, GFAP: glial fibrillary acidic protein, GPx: glutathione peroxidase, GSH: glutathione, ICAM-1: intercellular adhesion molecule 1, IL-1β: interleukin-1β, MAPK: mitogen-activated protein kinase, IL-8: interleukin-8, MDA: malondialdehyde, Nrf2: nuclear factor erythroid 2-related factor-2, PKC-β: protein kinase C-β, RGC-5: retinal ganglion cell 5, ROS: reactive oxygen species, SOD: superoxide dismutase, STZ: streptozotocin, T1DM: type 1 diabetes mellitus, TBARs: thiobarbituric acid reactive substances, TNF-α: tumor necrosis factor alfa, TrkB: tropomyosin related kinase B, VEGF: vascular endothelial growth factor, ↑: increase, ↓ decrease.

**Table 4:** Effects of flavones on cellular and molecular mechanisms affected by diabetic retinopathy.

Name of Substances/Compound	Dose	Model	Observations	Reference
Baicalein	75, 150 and 300 mg/kg orally administered for 24 weeks	STZ-induced T1DM rats	↓ microglial activation and IL-18, TNF-α and IL-1β expression	[81]
			↓ GFAP and VEGF expression in Müller cells	
			↓ vascular abnormality and ganglion cell loss	
	75 mg/kg in drinking water	Ins2^Akita^ mice	↑ pSHP1 levels	[83]
			↓ HETE, ICAM-1, VCAM-1 and IL-6 levels	
			↓ ROS generation, and NOX2 expression	
			↓ pVEGF-R2 levels	
Baicalin	2.5, 5, 10, 50 and 100 μM for 12 h	High glucose-exposed ARPE-19 and HRMECs	↑ miR-145 levels	[84]
			↑ cell proliferation	
			↓ apoptosis	
			↓ IL-1β, IL-6 and IL-8 release and ROS levels	
			↓ NF-κB and p38 MAPK pathways	
Scutellarin	0.1, 1, 10 or 100 nM; 0,1 or 1 μM for 48 h	High glucose and hypoxia-mimetic agent-exposed HRECs	↓ cell proliferation, migration, and tube formation	[90]
			↓ VEGF levels	
			↓ HIF-1α protein and mRNA levels	
			↓ NADPH oxidase activity	
	20 and 50 μM for 6 h	High glucose-exposed BV-2 cells	↓ NF-κB and TNF-α expression	[89]
			↓ microglia cell activation	
			↓ BRB damage	
			↓ ERK1/2 phosphorylation	
	20 and 50 μM for 6 h	TNF-α-exposed HRECs and ARPE-19 cells	↑ claudin-1 and claudin-19 expression	
			↑ Nrf2 nuclear accumulation	
			↓ ROS formation and BRB damage	
	5 and 10 mg/kg intragastric administration for 1 month	STZ-induced T1DM rats	↑ claudin-1 and claudin-19 expression	
			↓ microglia cell activation	
			↓ BRB breakdown	
			↓ ERK1/2 phosphorylation	
Nepetin	2.5, 5 and 10 µM for 24 h	IL-1β-exposed ARPE-19 cells	↓ IL-6, IL-8 and MCP-1 levels	[91]
			↓ nuclear translocation of NF-κB p65	
			↓ phosphorylation of inhibitor of nuclear factor kappa B and IκB kinase	
			↓ phosphorylation of ERK1/2, JNK and p38 MAPK	
Silybin	15 and 30 mg/kg orally administered for 22 weeks	STZ and high-fat diet-induced T2DM rats	↓ obliterated retinal capillaries	[92]
			↓ leukostasis and ICAM-1 levels	
Chrysin	1, 10 and 20 µM for 3 days	Glucose-exposed RPE cells	↑ PEDF levels	[93]
			↓ VEGF and IGF-1 levels	
			↓ AGE secretion and RAGE induction	
			↓ ER stress	
	1, 10 and 20 µM for 3 days	AGE-BSA-exposed RPE cells	↑ PEDF, RPE65, LRAT and RDH5 levels	
			↓ ER stress	
	10 mg/kg orally administered for 10 weeks	db/db mice	↑ ONL thickness	
			↑ RPE65, LRAT, RDH5, CRBP, CRALBP, IRBP, STRA6 and rhodopsin levels	
			↓ AGE secretion and RAGE induction	
			↓ ER stress	
Diosmin	100 mg/kg intragastric administration	Retinal ischemia/reperfusion in rats	↑ SOD, GPx and CAT activities	[97]
			↑ retinal a- and b-wave amplitudes	
			↑ INL, IPL, ONL and total retinal thicknesses	
			↑ ganglion cells number	
			↓ edema	
			↓ MDA	
			↑ retinal a- and b-wave amplitudes	[96]
			↑ ZO-1 and occludin levels	
			↓ VEGF levels	
			↓ Evans blue leakage	
	0.1, 1 and 10 µg/mL	High glucose-exposed ARPE-19 cells	↑ cell viability	[98]
			↑ Bcl-2/Bax	
			↓ capase-3 activity and cytochrome c release	
			↓ ROS levels	
			↓ JNK and p38 phosphorylation	

AGE: advanced glycation end products, ARPE-19: human retinal pigment epithelial cell line, Bax: Bcl-2 associated X protein, Bcl-2: B cell lymphoma-2 protein, BRB: blood–retinal barrier, BSA: bovine serum albumin, CAT: catalase, CRALBP: cellular retinaldehyde-binding protein-1, CRBP: cellular retinol-binding protein, ER: endoplasmic reticulum, ERK1/2: extracellular signal-regulated protein kinases 1 and 2, GFAP: glial fibrillary acidic protein, GPx: glutathione peroxidase, HETE: hydroxyeicosatetraenoic acids, HIF1-α: hypoxia inducible factor 1α, HRECs: human retinal endothelial cells, HRMECs: human retinal microvascular endothelial cells, ICAM-1: intercellular adhesion molecule 1, IGF-1: insulin-like growth factor-1, IκB kinase: inhibitor of κB kinase, IL-1β: interleukin-1β, IL-6: interleukin-6, IL-8: interleukin-8, IL-18: interleukin-18, INL: inner nuclear layer, Ins2^Akita^, genetic animal model of T!DM, IPL: inner plexiform layer, IRBP: interphotoreceptor retinoid-binding protein, JNK: c-Jun N-terminal kinases, LRAT: lecithin retinol acyltransferase, MAPK: mitogen-activated protein kinase, MCP-1: monocyte chemoattractant protein, MDA: malondialdehyde, miR-145, microRNA-145, NADPH: Nicotinamide adenine dinucleotide phosphate, NF-κB: nuclear factor kappa-B, NF-κB p65: NF-κB p65 subunit, Nox2: NADPH oxidase 2, Nrf2: nuclear factor erythroid 2-related factor-2, ONL: outer nuclear layer, PEDF: pigment epithelium-derived factor, pVEGF-R2, phosphorylated VEGF receptor 2, RAGE: AGE receptor, RDH5: retinol dehydrogenase 5, ROS: reactive oxygen species, RPE: retinal pigment epithelium, SOD: superoxide dismutase, STRA6: stimulated by retinoic acid 6, STZ: streptozotocin, T1DM: type 1 diabetes mellitus, T2DM: type 2 diabetes mellitus TNF-α: tumor necrosis factor alfa, VCAM-1: vascular cell adhesion molecule-1, VEGF: vascular endothelial growth factor, ZO-1: zonula occludens-1, ↑: increase, ↓ decrease.

**Table 5:** Effects of flavonols on cellular and molecular mechanisms affected by diabetic retinopathy.

Name of Substances/Compound	Dose	Model	Observations	Reference
Kaempferol	5 and 25 μM for 24 h	High glucose-stimulated HRECs	↓ VEGF and PGF mRNA levels	[100]
			↓ cell proliferation, migration, migration distance and sprouting of HRECs	
			↓ PI3K expression and ERK1/2, Src, and Akt1 activation	
	10, 15, 20, 25, 30 and 35 µM	N/A	↓ α-glucosidase and α-amylase activities	[101]
Quercetin	25 and 50 mg/kg orally administered for 24 weeks	STZ-induced T1DM rats	↑ GSH levels	[103]
			↑ SOD and CAT activities	
			↑ ganglion cells number and retinal thickness	
			↓ TNF-α and IL-1β levels	
			↓ NF-kB and caspase-3 levels	
			↓ GFAP and aquaporin-4 levels	
	150 mg/kg by intragastric injection for 20 weeks	STZ-induced T1DM rats	↓ MMP-9 and VEGF serum levels	[102]
			↓ MMP-9 and VEGF RNA and protein levels	
	10, 20, 30, 40 and 50 μM for 24 h	High glucose-exposed ARPE-19 cells	↑ CyclinD1, CDK4 and Bcl-2 levels	[104]
			↑ MiR-29b expression	
			↑ PTEN/AKT pathway	
			↓ NF-κB pathway via a miR-29b-dependent way	
			↓ viability loss, apoptosis, MCP-1 and IL-6 production and ROS generation	
			↓ p53, Bax and cleaved-caspase-3 expression	
Rutin	100 mg/kg orally administered for 5 weeks	STZ-induced T1DM rats	↑ blood insulin levels	[106]
			↑ Bcl-2 levels	
			↑ BDNF, NGF and GSH	
			↓ blood glucose levels	
			↓ TBARs	
			↓ caspase-3 levels	
	50 mg/kg orally administered for 24 weeks	STZ-induced T1DM rats	↑ total antioxidant capacity of the retinas	[107]
			↓ VEGF, TNF-α and aldose reductase protein levels	
			↓ vascular leakage of fluorescein	
Galangin	20 and 50 μM for 6 h	D-glucose-stimulated microglial BV2 cells	↓ BRB damage	[108]
			↓ ROS formation	
			↓ microglia cells activation	
			↓ ERK1/2 phosphorylation	
			↓ NF-κB and Egr1 protein levels	
			↓ TNF-α levels	
	20 and 50 μM for 6 h	TNF-α-exposed HRECs and ARPE-19 cells	↑ claudin-1 and occludin levels	
			↑ Nrf2 activation	
			↓ BRB damage	
			↓ ROS formation	
	1 and 10 mg/kg injection for 1 month	STZ-induced T1DM mice	↓ BRB damage	
			↓ ROS formation	
			↓ microglia cells activation	
			↓ ERK1/2 phosphorylation, NF-κB Egr1 protein	
			↓ TNF-α levels	
Myricetin	N/A	AGE-BSA-exposed bovine retinal pericytes	↓ pericytes migration	[109]
			↓ ERK1/2-FAK-1-paxillin phosphorylation	
	5 or 10 mM intravitreally injection	AGE-BSA-intravitreally injected rats	↓ pericytes migration	
			↓ ERK1/2-FAK-1-paxillin phosphorylation	
	2.5, 5, 10, 20 and 40 μg/mL for 4 h	Glucose oxidase-exposed ARPE-19 cells	↑ Nrf2 and SOD2 levels	[110]
			↓ production of H_2_O_2_ and intracellular ROS	
			↓ nitric oxide producer transcription	
	0.02, 0.2, 2, 20 and 40 μg/mL for 48 h	High glucose-exposed ARPE-19 cells	↑ antioxidant proteins and other protective factors	[111]
			↑ Nrf2 pathway	
			↓ intracellular ROS levels and	
			AGE formation	
			↓ NFkB1 expression	
			and RAGE	
Icariin	5 mg/kg orally administered for 12 weeks	STZ-induced T1DM rats	↑ RECA, Thy-1, Brn3a, and Collagen IV and CA-II levels	[112]
			↑ VEGF levels	
	0, 10, 100 and 1000 nmol/mL for 3 days	RGC cells from control and diabetic rats	↑ neurite growth	

AGE: advanced glycation end products, AKT: protein kinase B, ARPE-19: human retinal pigment epithelial cell line, Bax: Bcl-2 associated X protein, Bcl-2: B cell lymphoma-2 protein, BDNF: brain-derived neurotrophic factor, BRB: blood–retinal barrier, Brn3a: brain-specific homeobox/POU domain protein 3A, BSA: bovine serum albumin, CA-II: carbonic anhydrase II, CAT: catalase, CDK4: cyclin-dependent kinase 4, Egr1: early growth response protein 1, ERK1/2: extracellular signal-regulated protein kinases 1 and 2, FAK-1: focal adhesion kinase 1, GFAP: glial fibrillary acidic protein, GSH: glutathione, H_2_O_2_: hydrogen peroxide, HRECs: human retinal endothelial cells, IL-1β: interleukin-1β, IL-6: interleukin-6, MCP-1: monocyte chemoattractant protein, miR-29b, microRNA-29b, MMP-9: metalloproteinase-9, N/A: not applicable, NF-κB: nuclear factor kappa-B, NFkB1, nuclear factor of kappa light polypeptide gene enhancer in B-cells 1, NGF: nerve growth factor, Nrf2: nuclear factor erythroid 2-related factor-2, PGF: placenta growth factor, PI3K: phosphoinositide 3-kinase, PTEN, phosphatase and tensin homolog deleted on chromosome 10, RAGE: AGE receptor, RECA: rat endothelial cell antigen, RGC: retinal ganglion cell, ROS: reactive oxygen species, SOD: superoxide dismutase, STZ: streptozotocin, T1DM: type 1 diabetes mellitus, TBARs: thiobarbituric acid reactive substances, TNF-α: tumor necrosis factor alfa, VEGF: vascular endothelial growth factor, ↑: increase, ↓ decrease.

**Table 6:** Effects of isoflavones on cellular and molecular mechanisms affected by diabetic retinopathy.

Name of Substances/Compound	Dose	Model	Observations	Reference
Biochanin A	10 and 15 mg/kg orally administered for 6 weeks	STZ-induced T1DM rats	↓ blood glucose levels	[114]
			↓ VEGF, TNF-α and IL-1β levels	
Formononetin	0.1, 0.5, 1, 5 and 10 µmol/L for 24 h	H_2_O_2_-exposed RGC-5 cells	↑ RGC-5 cell viability	[115]
			↑ MnSOD activity	
			↓ superoxide anions, MDA and 8-OHdG levels	
			↓ apoptosis and NF-κB pathway activation	
Puerarin	6 intraperitoneal injections80 mg/kg	STZ-induced T1DM rats	↓ morphological changes of INL and ONL	[116]
			↓ VEGF and HIF-1α mRNA	
	140 mg/kg	RPE cells from STZ-induced T1DM rats	↓ apoptosis	[121]
			↓ NT, C3, iNOS mRNA expression	
			↓ Fas/FasL	
	Low and high doses for 4 weeks	STZ-induced diabetic rats	↑ ONL thickness (high-dose puerarin)	[117]
			↓ VEGF levels	
			↓ AGEs accumulation (high-dose puerarin)	
			↓ RAGE levels (high-dose puerarin)	
	125, 250 and 500 mg/kg intragastric administered for 4 weeks	STZ-induced T1DM rats	↑ retinal b-wave amplitude	[120]
			↓ retinal cell apoptosis	
			↓ NF-κB p65 activity	
	500 mg/kg intragastric administration for 4 weeks	STZ-induced T1DM rats	↑ SOD activity	[119]
			↓ MDA content	
			↓ RAGE and VEGF levels	
	1, 5 and 10 µM for 1 h	AGE-BSA-exposed bovine retinal pericytes	↓ apoptosis	[123]
			↓ ROS generation and NADPH oxidase activity	
			↓ p47phox and Rac1 phosphorylation	
			↓ NF-kB activation	
	10 µM for 1 h	Intravitreal injection of AGE-modified rat serum albumin rats	↓ apoptosis of the retinal pericyte of rats	
	140 mg/kg intraperitoneal injection for 60 (56) days	RPE cells from STZ-induced T1DM rats	↓ apoptosis	[122]
			↓ NT and iNOS mRNA levels	
			↓ Fas/FasL protein expression	
	10, 25 and 50 µM for 24 h	IL-1β-exposed TR-iBRB2 cells	↓ leukostasis and apoptosis	[124]
			↓ VCAM-1 and ICAM-1 expression	
			↓ mitochondrial dysfunction	
	10^−7^,10^−6^,10^−5^ and 10^−4^ mol/L for 36 h	NMDA-exposed RGCs cells	↑ SOD and NO production	[125]
			↑ Bcl-2 expression	
			↓ RGCs injury	
			↓ ROS and MDA levels	
			↓ nNOS and iNOS expression	
			↓ Bax expression and caspase-3 activity	
			↓ JNK and p38 phosphorylation	
	10^−7^,10^−6^,10^−5^ and 10^−4^ mol/L for 36 h	NMDA-intravitreally injected rats	↓ RGCs loss	
	250 and 500 mg/kg intragastric administration for 4 weeks	STZ-induced T1DM rats	↑ insulin levels	[118]
			↑ retinal b-wave amplitude	
			↑ SOD activity	
			↓ blood glucose levels	
			↓ MDA	
			↓ STAT3 mRNA and protein levels	
3,6,7,40,50-pentamethoxy-5,30-dihydroxyflavone	160 mg/kg for 8 weeks	STZ (35 mg/kg)-induced DM rats	↓ aldose reductase, SOD, CAT, and GPx and GSH levels	[126]
			↓ TBARs and protein carbonyl levels	
			↓ sorbitol accumulation	

8-OHdG: 8-hydroxy-2-deoxyguanosine, AGE: advanced glycation end products, Bax: Bcl-2 associated X protein, Bcl-2: B cell lymphoma-2 protein, BSA: bovine serum albumin, C3: complement 3, CAT: catalase, DM, diabetes mellitus, Fas: tumor necrosis factor receptor superfamily member 6, FasL: Fas ligand, GPx: glutathione peroxidase, GSH: glutathione, H_2_O_2_: hydrogen peroxide, HIF1-α: hypoxia inducible factor 1α, ICAM-1: intercellular adhesion molecule 1, IL-1β: interleukin-1β, INL: inner nuclear layer, iNOS: inducible NOS, JNK: c-Jun N-terminal kinases, MDA: malondialdehyde, MnSOD: manganese-dependent superoxide dismutase, NADPH: Nicotinamide adenine dinucleotide phosphate, NF-κB: nuclear factor kappa-B, NF-κB p65: NF-κB p65 subunit, NMDA: *N*-methyl-D-aspartic acid, nNOS: neuronal NOS, NO: nitric oxide, NT: nitrotyrosine, ONL: outer nuclear layer, p47phox, neutrophil cytosol factor 1, Rac1, Ras-related C3 botulinum toxin substrate 1, RAGE: AGE receptor, RGC-5: retinal ganglion cell 5, ROS: reactive oxygen species, RPE: retinal pigment epithelium, SOD: superoxide dismutase, STAT3, signal transducer and activator of transcription, STZ: streptozotocin, T1DM: type 1 diabetes mellitus, TBARs: thiobarbituric acid reactive substances, TNF-α: tumor necrosis factor alfa, TR-iBRB2: retinal capillary endothelial cells, VCAM-1: vascular cell adhesion molecule-1, VEGF: vascular endothelial growth factor, ↑: increase, ↓ decrease.

**Table 7:** Clinical studies with flavonoids effect in diabetes and diabetic retinopathy (DR).

Study	Participants	Dose	Duration	Results	Reference
Contribution of flavonoid intake for decreasing the risk of development chronic diseases	*n* = 10,054, of which 546 were addressed to measure the risk of developing diabetes	N/A	1 year	↓ risk of developing T2DM associated with higher quercetin and myricetin intake	[127]
Green tea catechins impact, with or without caffeine, on glycemic control markers	*n* = 1584 out of twenty-two eligible trials	N/A	N/A	↓ FBG levels	[128]
				No significant difference in FBI, HbA1c and HOMA-IR	
Orally administered combination of flavonoids with *Centella asiatica* and *Melilotus* effect for the treatment of CME without macular thickening	*n* = 70 with T2DM and CME without macular thickening	Oral combination of 300 mg diosmin, 15 mg *C. asiatica* and 160 mg *Melilotus* per day	36 months	↑ retina sensitivity	[129]
				No differences in visual acuity, mean central retinal thickness, stability fixation, HbA1c percentage, microalbuminuria and blood pressure	
Purified anthocyanins effect to increase serum adiponectin	*n* = 160 prediabetic or newly diagnosed diabetic	2 × 320 mg per day	12 weeks	Anthocyanins supplementation increased serum adiponectin and decreased fasting glucose in newly diagnosed diabetics but not in prediabetic patients	[130]
Chinese green tea consumption and the risk of diabetic retinopathy	*n* = 100 with DR	Drink Chinese green tea for at least once a week	1 year	↓ 50% risk of developing DR in people who regularly drink green tea than those who do not	[132]
	*n* = 100 diabetic without DR				
Pycnogenol^®^ effect on the progression of visual acuity	*n* = 1169 with T1DM and T2DM that presented DR	60–120 mg per day	6 months	↓ progression of visual loss	[133,134]
				No significant improvements in patients’ sight	
Effect of Pycnogenol^®^ in early stages of DR	*n* = 46 diagnosed with moderate degree of diabetic macular edema	150 mg daily	2 months	Visual and baseline improvement	[134]
Flavonoid-rich diet impact on DR and diabetes-related biomarkers	*n* = 381 diabetic patient from NHANES 2003–2006	N/A	N/A	↓ risk of developing DR by 30%	[131]
				↓ C-reactive protein levels, HbA1C and glucose	
Effects of antioxidant supplementation in ROS circulating levels and in changes in CMT	*n* = 68 with NPDR	One tablet containing 50 mg of pycnogenol, 30 mg of Vitamin E and 20 mg of CoQ per day	6 months	↓ ROS levels and CMT	[135]

CME: cystoid macular edema, CMT: central macular thickness, CoQ, coenzyme Q10, DR: diabetic retinopathy, FBG: fasting blood glucose, FBI: fasting blood insulin, HbA1c: glycated hemoglobin, HOMA-IR: homeostatic model assessment of insulin resistance, N/A: not applicable, NHANES: National Health and Nutrition Examination Survey, NPDR: non-proliferative diabetic retinopathy, ROS: reactive oxygen species, T1DM: type 1 diabetes mellitus, T2DM: type 2 diabetes mellitus, ↑: increase, ↓ decrease.
